# Evaluation of a New Portable High-Definition Video Laryngoscope (Tuoren VLHM5A) for Tracheal Intubation in Adults: Technical Report and Service Assessment

**DOI:** 10.7759/cureus.109204

**Published:** 2026-05-19

**Authors:** Giuseppe Pascarella, Alessandro Strumia, Sabrina Migliorelli, Felice E Agrò

**Affiliations:** 1 Anesthesia and Intensive Care, Fondazione Policlinico Universitario Campus Bio-Medico, Rome, ITA

**Keywords:** airway management, anesthesia, difficult airway, tracheal intubation, videolaryngoscopy

## Abstract

Videolaryngoscopy (VL) is increasingly recommended as the primary technique for tracheal intubation, supported by major international guidelines, which endorse VL for routine use to provide improved glottic visualization, higher first attempt success, and reduced complications, including failed intubation and airway trauma. However, despite these advantages, VL adoption remains low due to higher cost, variations in devices, training limitations, and restricted availability. This technical report summarizes early institutional experience with the Tuoren VLHM5A - a new portable, high-definition (HD) VL system - used in 50 elective surgical patients. The device demonstrated excellent visualization, rapid intubation, and 100% first-attempt success in both “normal” and “predicted difficult” airways when used by experienced anesthesiologists in this preliminary experience. However, these findings should be interpreted with caution given the limited sample size and the absence of a comparator device. If confirmed in larger studies, portable HD VL systems may help address some of the barriers to wider VL adoption and support guideline-based airway management.

## Introduction

Videolaryngoscopy (VL) has reshaped contemporary airway management, offering superior glottic visualization, a higher first successful attempt rate, and fewer intubation-related complications (i.e. failed intubation and airway trauma) compared with direct laryngoscopy (DL). Improved visualization facilitates faster identification of the glottic opening and more controlled advancement of the endotracheal tube, potentially reducing repeated attempts, airway trauma, and hypoxemia during airway management. International guidelines, including the 2025 European consensus on universal VL implementation and the Canadian Airway Focus Group recommendations, highlight VL as the preferred technique for routine tracheal intubation when available [1,2]. These recommendations are supported by extensive literature demonstrating reduced airway trauma, fewer esophageal intubations, and improved patient safety [3]. Despite these well-established benefits, adoption of routine VL across healthcare systems remains inconsistent [4]. The barriers to adoption include equipment cost, heterogeneity among devices, inadequate training opportunities, and resistance to change from traditional DL techniques [1]. Limited VL availability outside the operating room - such as in emergency departments, intensive care units, and prehospital environments - further restricts its universal use. This report presents our institutional experience using a new high-definition portable videolaryngoscope in 50 elective surgical patients and evaluates its performance across normal and predicted difficult airways.

## Technical report

**Figure 1:**
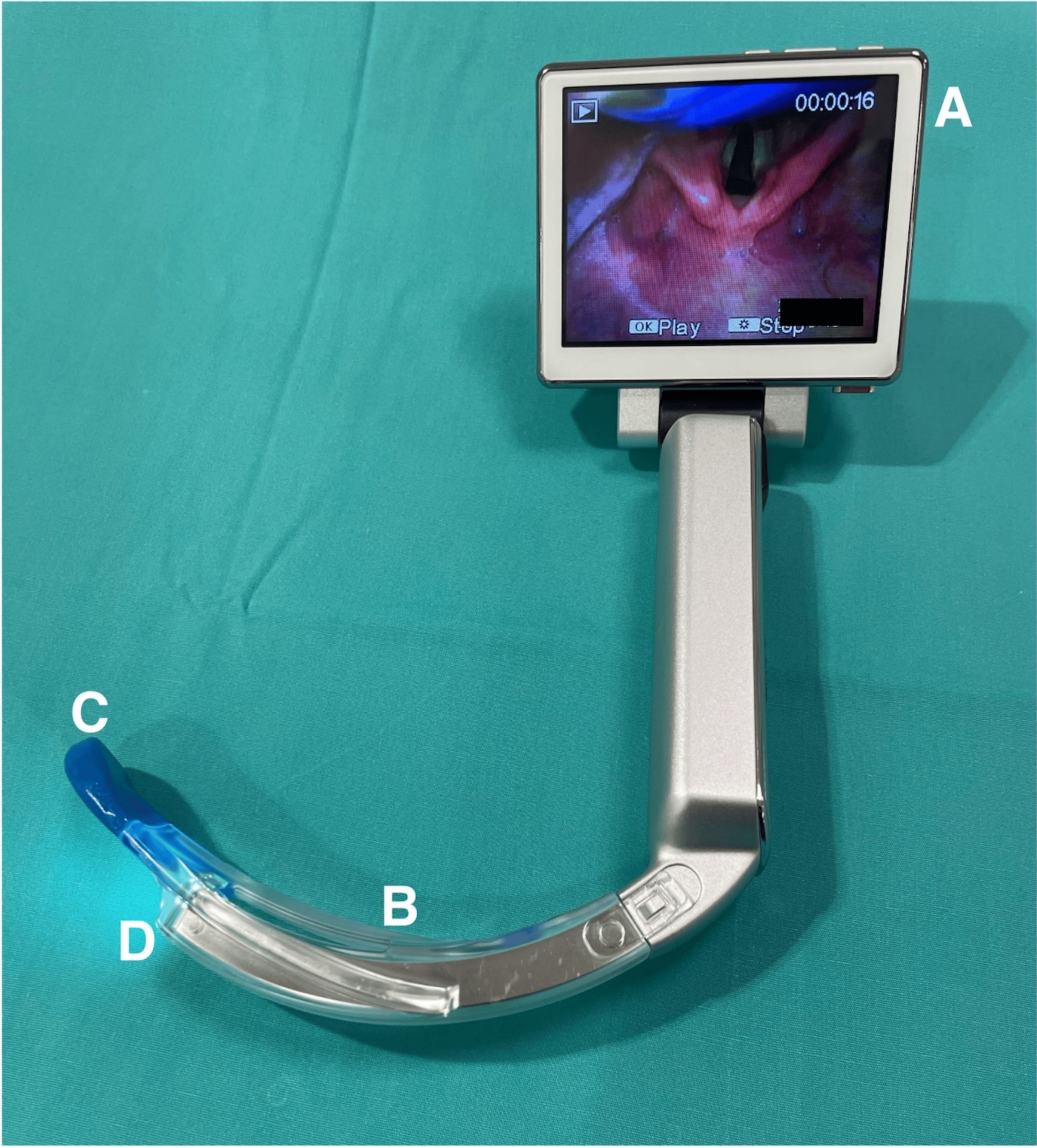
Main Characteristics of Tuoren VLHM5A Videolaryngoscope A. 3.5” HD monitor (three million pixels); B. Disposable anti-fog blade; C. Atraumatic blade tip in soft silicone; D. 50-60° Field angle camera

The Tuoren VLHM5A videolaryngoscope (Tuoren Medical, Changyuan, Henan, China) was introduced at our institution to support broader use of portable VL in everyday clinical practice (Figure 1).

This device belongs to the category of videolaryngoscopes with an intermediate blade angulation - approximately 45° - while still providing a wide visual field of up to 60°. Devices in this class aim to combine some of the advantages of hyper‑angulated blades with improved ease of tube delivery, typically associated with standard geometry blades. The VLHM5A also features a high-definition three-million-pixel monitor, anti-fog disposable blades, optical quartz-glass lens, a 50-60° field angle, atraumatic silicone tip, and ≥200 minutes of operating autonomy. These characteristics make it a practical choice for routine cases, difficult airways, and teaching environments.

This technical evaluation represents a service assessment of a commercially available videolaryngoscope used in routine clinical practice at our institution. The implementation of this device was approved by the Head of the Department, and clinical data were collected retrospectively from anonymized anesthetic records. According to institutional policies for service evaluations, formal ethics committee approval was not required.

This experience included 50 adult patients undergoing elective surgery requiring general anesthesia and tracheal intubation at our institution between May and September 2025. Given the descriptive nature of this service evaluation and the absence of a comparator group, no inferential statistical analyses were performed. Continuous variables are reported as mean±standard deviation, and categorical variables as number and percentage. Forty patients had no difficult airway predictors, while 10 exhibited predicted difficulty, according to an El-Ganzouri Risk Index (EGRI) score (Table 1) [5]. The EGRI is a composite airway assessment score based on seven preoperative predictors of difficult intubation, generating a total score from 0 to 12; scores ≥4 are typically associated with an increased risk of difficult tracheal intubation [5].

**Table 1:** Characteristics of Patients Patients were classified as having a normal airway or a predicted difficult airway according to the El-Ganzouri Risk Index (EGRI). An EGRI score ≥4 was considered indicative of predicted difficult tracheal intubation. Airway assessment variables included mouth opening, ability to prognath, thyromental distance, Mallampati classification, and neck extension. Values are presented as number of patients (%) or mean±standard deviation. Mallampati classification: a preoperative airway assessment based on the visibility of oropharyngeal structures, graded as follows: Class I, full visibility of soft palate, uvula, fauces, and pillars; Class II, visibility of soft palate, uvula, and fauces; Class III, visibility of soft palate and base of uvula; and Class IV, only hard palate visible.

	Normal Airway (n = 40)	Predicted Difficult Airway (n = 10)
Age (years)	48±15	52±12
Sex (M/F)	18/22	7/3
Weight		
<90 kg	36 (90%)	4 (40%)
90-100 kg	3 (7.5%)	4 (40%)
100 kg	1 (2.5%)	2 (20%)
Mouth opening		
≥4 cm	39 (37.5%)	9 (90%)
<4 cm	1 (2.5%)	1 (10%)
Inability to perform mandibular protrusion	2 (5%)	2 (20%)
Thyromental distance		
>6.5 cm	35 (87.5%)	2 (20%)
6-6.5 cm	4 (10%)	6 (60%)
<6 cm	1 (2.5%)	2 (20%)
Mallampati classification		
I	20 (50%)	1 (10%)
II	17 (42.5%)	4 (40%)
III-IV	3 (7.5%)	5 (50%)
Neck extension		
>90°	38 (95%)	6 (60%)
80-90°	2 (5%)	3 (30%)
<80°	0%	1 (10%)
Previous difficult intubation	0%	0%

All procedures were part of routine care, and data were collected anonymously. Patients underwent three minutes of preoxygenation with 100% oxygen via a tight-fitting face mask. General anesthesia was induced with propofol 2 mg/kg, fentanyl 3 mcg/kg, and rocuronium 0.6 mg/kg. Intubation was performed using a mandrinated endotracheal tube; the stylet was shaped to mirror the VL blade curvature to optimize maneuverability. The endotracheal tube cuff was inflated under direct video visualization to ensure correct positioning and avoid mucosal injury. All the tracheal intubations were performed by anesthetists with almost 5 years of experience with videolaryngoscopy.

**Figure 2:**
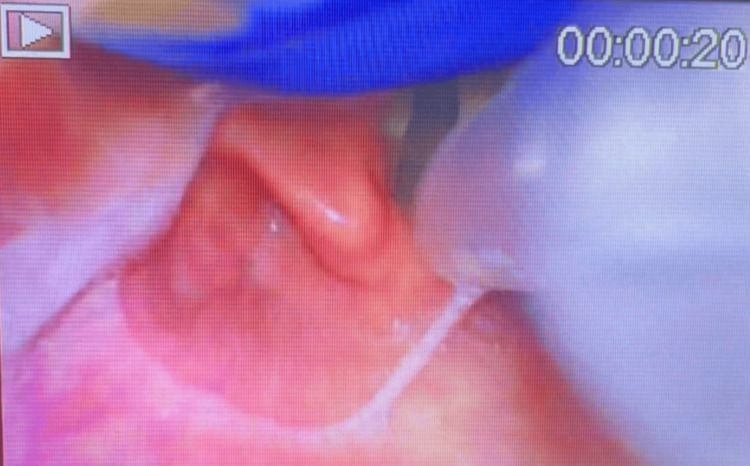
Tracheal intubation through Tuoren VLHM5A Videolaryngoscope

The videolaryngoscopy and tracheal intubation sequence was standardized using a four-step technique [6]: first, the operator looks into the patient’s mouth while gently inserting the blade in the midline, sliding over the center of the tongue; the clinician then shifts their gaze to the screen, advancing the blade until the glottic plane is clearly identified; subsequently, the operator returns their gaze to the mouth to correctly introduce the tracheal tube with the stylet; finally, the clinician returns to the screen to guide the tube tip through the glottis under continuous video visualization (Figure 2).

The quality of glottic visualization was assessed using an 11-point Numeric Rating Scale (NRS; range 0-10), reflecting the operator’s overall perception of image quality during videolaryngoscopy. We choose this measurement to quantify a respondent's perceived experience on a numerical range [7].

On this scale, 0 corresponded to the worst glottic visual quality reasonably expected from a videolaryngoscope, whereas 10 represented the best possible visual quality. For descriptive and reporting purposes, NRS values were categorized as follows: 0-3=bad, 4-6=good, and 7-10=excellent. This approach was adopted to provide a clinically intuitive interpretation of visual quality while maintaining the granularity of the original numeric assessment.

First-pass success was obtained 100% in all patients (50/50; 95% confidence interval (CI) 92.9-100%). Mean intubation time was 35±10 seconds in normal airways and 42±16 seconds in predicted difficult airways. All cases achieved Cormack-Lehane grade I or II visualization [8], and no external laryngeal manipulations were required. Moreover, no operators reported technical difficulties during videolaryngoscopy or tracheal intubation, including blade handling, glottic visualization, or tube advancement. No complications - such as bleeding, dental injury, airway trauma, or postoperative dysphonia - were observed (Table 2).

**Table 2:** Intubation Performance Values are presented as numbers of patients (%) and mean±standard deviation. Cormack-Lehane grade: classification of laryngeal view obtained during direct laryngoscopy, graded as follows: Grade I, full view of the glottis; Grade II, partial view of the glottis; Grade III, only epiglottis visible; and Grade IV, neither glottis nor epiglottis visible.

Outcomes	Normal Airway (n=40)	Predicted Difficult Airway (n=10)
First pass success	40 (100%)	10 (100%)
Time to intubation (seconds)	35±10	42±16
Time to glottic visualization (seconds)	17.2±4.5	22.4±6.3
Quality of glottic view		
Bad	0%	0%
Good	8 (20%)	2 (20%)
Excellent	32 (80%)	8 (80%)
Cormack-Lehane grade		
I	36 (90%)	8 (80%)
II	4 (10%)	2 (20%)
III-IV	0%	0%
External laryngeal manipulation	0%	0%
Bleeding	0%	0%
Postoperative dysphonia	0%	0%

## Discussion

The results of this evaluation support the routine use of high-definition portable videolaryngoscopy in airway management, highlighting the device’s reliability and ease of use. The VLHM5A demonstrated excellent laryngeal visualization, rapid intubation, and 100% first-pass success in both normal and predicted difficult airway cases when used by experienced anesthesiologists, aligning with the recommendations of major airway management guidelines. 

The overall intubation performance was even better than a previous experience conducted in our institution with other devices [9]. Improved visualization, reduced airway trauma, and enhanced team communication through shared screen viewing are among the well-recognized benefits of VL. Although the VLHM5A does not incorporate a hyper‑angulated blade, it nevertheless allowed smooth and successful tracheal intubation in all patients, including those with predicted airway difficulty. This observation reflects findings from the broader literature, which does not demonstrate the clear superiority of hyper‑angulated blades over intermediate‑angled devices in most clinical settings. Not by chance, the Canadian guidelines recommend switching from a hyper‑angulated blade to a non‑hyperangulated one when difficulty with tube delivery arises during videolaryngoscopy, reinforcing the concept that intermediate‑angled devices may offer a favorable balance between visualization and ease of intubation [2].

Several barriers to widespread VL adoption have been highlighted in international consensus statements, including equipment cost, limited device availability and portability, inadequate training, and resistance to transitioning from DL. The VLHM5A addresses many of these challenges through its portability, ease of use, and high-definition imaging. These characteristics make it particularly valuable in resource-limited settings and in environments where rapid deployment of airway equipment is necessary.

The atraumatic design, anti-fog capability, and reliable image quality observed in this evaluation reinforce the device’s suitability for both routine intubation and predicted difficult airway scenarios. The effectiveness of shaping the stylet to match the blade curvature further enhances maneuverability and may help mitigate common challenges in VL-guided intubation [10]. Looking ahead, an important future direction will be evaluating the VLHM5A’s utility in awake tracheal intubation. Awake VL techniques are increasingly recognized as effective alternatives to fiberoptic bronchoscopy in selected patients, particularly when airway anatomy or pathology favors indirect visualization [3]. Given the device’s excellent imaging performance, assessing its role in awake intubation protocols represents a meaningful next step in expanding its clinical applicability.

To date, this is the first report describing the clinical use of the VLHM5A videolaryngoscope; however, several limitations should be acknowledged. The sample size was relatively small and the evaluation was conducted in a single institution, which may limit the generalizability of the findings. In addition, all intubations were performed by anesthesiologists experienced in videolaryngoscopy, potentially introducing operator-related bias. Airway visualization was documented according to our institutional protocol using the Cormack-Lehane classification rather than the Percentage of Glottic Opening (POGO) score, which may be more specifically suited to videolaryngoscopy; however, both scoring systems are subject to inter-operator variability. Furthermore, part of the evaluation relied on subjective assessments, including the use of NRS to describe video image quality, which is not a validated scoring system for videolaryngoscopy. Finally, this descriptive experience did not include a comparator device, formal statistical comparisons, or a prospective trial design. Therefore, these findings should be interpreted cautiously and confirmed in larger comparative studies.

## Conclusions

Tuoren VLHM5A videolaryngoscope, the glottic visualization tool, was evaluated in our anesthesia department. We found it to be a reliable and effective tool for routine tracheal intubation, achieving excellent visualization in the majority of cases and 100% first-pass success in both normal and predicted difficult airways. Its portability, ease of use, and high-definition imaging suggest a potential for widespread clinical use. However, further studies are warranted to better define the role of this device in clinical practice. In particular, prospective comparative studies with other videolaryngoscopes, evaluations in different clinical settings such as emergency and intensive care environments, and assessments involving operators with varying levels of experience would be valuable. Additional research may also explore its performance during awake tracheal intubation and its potential role in airway management training.

## References

[1] Gómez-Ríos MÁ, Van Zundert AA, McNarry AF (2025). Guidelines on strategies for the universal implementation of videolaryngoscopy. Eur J Anaesthesiol.

[2] Law JA, Duggan LV, Asselin M (2021). Canadian Airway Focus Group updated consensus-based recommendations for management of the difficult airway: part 1. Difficult airway management encountered in an unconscious patient. Can J Anaesth.

[3] Apfelbaum JL, Hagberg CA, Connis RT (2022). 2022 American Society of Anesthesiologists practice guidelines for management of the difficult airway. Anesthesiology.

[4] Orrock JL, Ward PA, McNarry AF (2024). Routine use of videolaryngoscopy in airway management. Int Anesthesiol Clin.

[5] Baby AE, D'souza MC, Krishnakumar M, Kavalakkatt DD (2024). Evaluating the predictive efficacy of the El-Ganzouri risk index for difficult laryngoscopy and intubation with King Vision(™) video laryngoscope: A prospective cohort study. Indian J Anaesth.

[6] Agrò FE, Doyle DJ, Vennari M (2015). Use of GlideScope® in adults: an overview. Minerva Anestesiol.

[7] Ponce de Leon S, Lara-Muñoz C, Feinstein AR, Wells CK (2004). A comparison of three rating scales for measuring subjective phenomena in clinical research. II. Use of experimentally controlled visual stimuli. Arch Med Res.

[8] Arkala A, Kaur M, Rauscher J, Carlson JN, Nikolla DA (2025). Reliability of the Cormack-Lehane classification: a scoping review. Cureus.

[9] Pascarella G, Caruso S, Antinolfi V, Costa F, Sarubbi D, Agrò FE (2020). The VL3 videolaryngoscope for tracheal intubation in adults: A prospective pilot study. Saudi J Anaesth.

[10] Cook TM (2024). Stylets, bougies and hyperangulated videolaryngoscopy. Anaesthesia.

